# Reflections as a settler person doing research in collaboration with Indigenous peoples.

**DOI:** 10.23889/ijpds.v7i3.1969

**Published:** 2022-08-25

**Authors:** Benjamin Harrap, Alison Gibberd, Melissa O'Donnell, Koen Simons, Sandra Eades

**Affiliations:** 1 The University of Melbourne; 2 University of South Australia

## Objectives

To reflect on my position as a settler person in Australia and ensure Indigenous voices are prioritised throughout my research, as part of a co-designed, Aboriginal-led study which aims to understand trends in the removal of Indigenous children born in Western Australia using data linkage and qualitative research.

## Approach

As a non-Indigenous person, it is important to reflect on my cultural background and acknowledge my limited understanding of the cultural context of the Indigenous communities represented in the data. Listening to Indigenous voices and collaborating with Indigenous peoples at all stages of my research – from my PhD supervisor to investigators on the broader study, to members of the community and policy reference groups – will be key to improve my understanding of the data from a system and context I am unfamiliar with.

## Results

Collaboration has been cyclical, with results from the qualitative research and discussion with the reference groups informing the initial quantitative research direction. Findings from this research were presented back to the groups, resulting in further questions and directions to explore.

The journey so far has been one of learning and understanding the skills I have and the role they can play whilst acknowledging the limits of my own knowledge and the need for Indigenous voices to guide the research in order to be doing research with Indigenous peoples, rather than on them.

## Conclusion

Co-design with Indigenous peoples is critical for doing research which affects them or uses data from their communities. Understanding my own cultural background and acknowledging the limitations of my experience continues to be important for honest and meaningful collaboration.

